# Anonymous fecal sampling and NIRS studies of diet quality: Problem or opportunity?

**DOI:** 10.1002/ece3.6354

**Published:** 2020-05-26

**Authors:** Luca Corlatti

**Affiliations:** ^1^ Chair of Wildlife Ecology and Management University of Freiburg Freiburg Germany

**Keywords:** chamois, individual heterogeneity, infrared spectroscopy, nonindependence, pseudoreplication, repeatability, *Rupicapra*

## Abstract

Investigating the drivers of diet quality is a key issue in wildlife ecology and conservation. Fecal near infrared reflectance spectroscopy (f‐NIRS) is widely used to assess dietary quality since it allows for noninvasive, rapid, and low‐cost analysis of nutrients. Samples for f‐NIRS can be collected and analyzed with or without knowledge of animal identities. While anonymous sampling allows to reduce the costs of individual identification, as it neither requires physical captures nor DNA genotyping, it neglects the potential effects of individual variation. As a consequence, regression models fitted to investigate the drivers of dietary quality may suffer severe issues of pseudoreplication. I investigated the relationship between crude protein and ecological predictors at different time periods to assess the level of individual heterogeneity in diet quality of 22 marked chamois *Rupicapra rupicapra* monitored over 2 years. Models with and without individual grouping effect were fitted to simulate identifiable and anonymous fecal sampling, and model estimates were compared to evaluate the consequences of anonymizing data collection and analysis. The variance explained by the individual random effect and the value of diet repeatability varied with seasons and peaked in winter. Despite the occurrence of individual variation in dietary quality, ecological parameter estimates under identifiable or anonymous sampling were consistently similar. This study suggests that anonymous fecal sampling may provide robust estimates of the relationship between dietary quality and ecological correlates. However, since the level of individual heterogeneity in dietary quality may vary with species‐ or study‐specific features, inconsequential pseudoreplication should not be assumed in other taxa. When individual differences are known to be inconsequential, anonymous sampling allows to optimize the trade‐off between sampling intensity and representativeness. When pseudoreplication is consequential, however, no conclusive remedy exists to effectively resolve nonindependence.

## Graphical abstract

Samples for dietary quality are often collected anonymously. Individual variation may entail severe issues of pseudoreplication. With low repeatability, anonymous sampling may provide robust estimates of the correlates of dietary quality. When pseudoreplication is consequential, no remedy exists to resolve nonindependence.

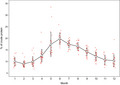

## 1 INTRODUCTION

Energy uptake has profound impacts on life history traits such as growth, survival, and reproduction (van Noordwijk & de Jong, 1986). Diet quality is a major components of animal nutrition (Barboza, Parker, & Hume, 2009), and investigating how internal and external factors can influence its variations is a key issue in wildlife ecology and conservation (Birnie‐Gauvin, Peiman, Raubenheimer, & Cooke, 2017). In particular, the occurrence of individual variation in nutritional processes has long been recognized (cf. VanValen, 1965), but attention to the importance of individual heterogeneity in wildlife studies of diet quality has been drawn only recently (Steyaert et al., 2012).

Dietary quality of free‐ranging animals is commonly assessed by noninvasive measurement of fecal nitrogen concentration (Leslie, Bowyer, & Jenks, 2008), either through chemical analyses (e.g., Gad & Shyama, 2011; Monteith, Monteith, Bowyer, Leslie, & Jenks, 2014) or near infrared reflectance spectroscopy (NIRS: Dixon & Coates, 2009; Kamler, Homolka, & Čižmár, 2004). NIRS analysis is based on the idea that the amount of near infrared radiation that is absorbed by C–H, N–H, and O–H bonds contains details on the chemical composition of food items, thus providing multiple indices of diet quality (Foley et al., 1998). As the quality of food consumed by animals can be highly variable in space and in time (e.g., Holand, 1994; Lurz, Garson, & Wauters, 2000), a high number of samples may be required to accurately represent diet quality variations. Fecal NIRS (f‐NIRS) allows for rapid and low‐cost analysis of multiple constituents of plant and animal tissues (Foley et al., 1998) and is arguably the most cost‐effective noninvasive technique for extensive, long‐term monitoring of dietary quality in wildlife populations (Garnick, Barboza, & Walker, 2018).

When samples for f‐NIRS analysis are genotyped or collected from animals that are captured and later tracked with Very High Frequency (VHF) or Global Positioning System (GPS) devices, dietary quality indices can be linked with specific individuals (Steyaert et al., 2012). If multiple samples per animal are collected, individual variation of the traits under study can be estimated (Hayes & Jenkins, 1997). In brown bear *Ursus arctos*, for example, individual heterogeneity alone explained about 22% of the variance in neutral detergent fiber (Steyaert et al., 2012). In regression analysis, individual heterogeneity in a given trait (the response variable) is most frequently estimated as the intraclass correlation coefficient (ICC: Wolak, Fairbairn, & Paulsen, 2012). ICC is defined as $\sigma_{\alpha }^{2}/\left(\sigma_{\alpha }^{2}+\sigma_{\varepsilon }^{2}\right)$ , where $\sigma_{\alpha }$ represents the variability of the trait among individuals and $\sigma_{\varepsilon }$ the variability of the trait within individuals (Nakagawa & Schielzeth, 2010). The proportion of among‐individual variance to the total variance of a trait is also known as repeatability (Hayes & Jenkins, 1997). In f‐NIRS studies, repeatability assesses how much dietary quality is consistent (cf. Harper, 1994): 0 when there is no clustering, that is, no pattern in diet quality within and between individuals, 1 when there is complete clustering, that is, diet quality is the same within individuals but different between individuals.

When individual heterogeneity occurs, it should be accounted for to secure robust estimates of f‐NIRS correlates (Steyaert et al., 2012), for example, by fitting individual random effects in multilevel models (Zuur & Ieno, 2016). Individual identification through physical captures or DNA analysis, however, may be costly and samples for f‐NIRS studies of dietary quality are often collected and analyzed on an anonymous basis, that is, without knowing the identity of the animals (e.g., Gad & Shyama, 2011; Halbritter & Bender, 2015). Although anonymous sampling allows to reduce the costs of identification, it neglects individual variation and may cause overrepresentation of some animals. Essentially, this reflects an issue of simple pseudoreplication; that is, the number of independent samples may be artificially inflated because multiple observations may have been taken on a single animal (Hurlbert, 1984; Millar & Anderson, 2004), possibly distorting the estimates of ecological correlates of dietary quality. However, neglecting individual heterogeneity, per se, does not necessarily lead to biased or variable results, and a multilevel modeling approach may be needed only when a consequential lack of independence occurs (cf. Corlatti, 2018).

No information is available about the consequences of unmodelled individual heterogeneity when fecal samples are collected and analyzed anonymously to study the drivers of wildlife dietary quality evaluated with f‐NIRS. In this paper, I aim to assess the level of individual heterogeneity (significance of individual grouping effect, *R* ^2^ and repeatability) when modeling the relationship between ecological variables and dietary quality of marked individuals of Alpine chamois *Rupicapra rupicapra rupicapra* monitored over two years. I then compare parameter estimates of models with and without individual grouping effect, thus simulating anonymization of fecal sampling. I finally discuss potential remedies for pseudoreplication when the aim is to investigate correlates of f‐NIRS dietary quality in wildlife studies.

## 2 MATERIALS AND METHODS

### 2.1 Study site

The study was conducted in 2011 and 2012 in the upper part of the Orco Valley, which extends over 10 km^2^ between 1,700 and 3,000 m a.s.l. within the Gran Paradiso National Park (Western Italian Alps, 45°26′30″N, 7°08′30″E). The area has a south‐facing slope dominated by colored fescue *Festuca varia*, and a north‐facing slope with woods of larch *Larix decidua* and patches of alder shrubs *Alnus viridis*. The climate in the study site is continental, with mean yearly rainfall of *c*. 1,000 mm and mean temperatures between −4°C in winter and 13°C in summer. The chamois in the Park has been protected since 1922 and, at the time of the study, the upper Orco Valley had a density of *c*. 20 chamois/km^2^.

### 2.2 Sample collection and f‐NIRS analysis

Twenty‐two adult male chamois were captured and marked with colored ear tags and GPS‐VHF collars, which collected 1 fix every 11 hr, except during the rut (6 November–5 December) when 1 fix every 3 hr was collected. Details about chamois captures and identification are reported in Corlatti et al. (2012) and in Corlatti, Lorenzetti, and Bassano (2019). All individuals were tracked and detected on a monthly basis between January 2011 and December 2012. One fresh fecal sample/month was collected for as many animals as possible immediately after deposition. Each sample was put in plastic bags linked with animal ID and collection date, and stored at −20°C until analysis (cf. Corlatti, 2018; Corlatti et al., 2019). Overall, 314 f‐NIRS samples were collected over the two years. Individual sample size ranged between 3 and 21, with mean ± *SD* =14.3 ± 5.6.

Fecal samples were dried in an oven (Memmert, Schwabach, Germany) at 60°C for 48 hr and ground with a grinder A11 basic (Ika). A subsample of feces (*n* = 86) was analyzed chemically with standardized methods for crude protein, crude fat, crude ash, and dry matter (Nehring, 1960) to calibrate the f‐NIRS analysis. Acid detergent fiber (ADF) and Lignin were determined by Van Soest detergent analyses (Otzelberger, 1983) and evaluated by cross validation (cf. Corlatti, Bassano, Valencak, & Lovari, 2013). A FT‐NIR Spectrometer MPA (Bruker Optik) equipped with software OPUS 5.5, the additional packages OPUS/LAB and OPUS/QUANT (2005, Bruker) and an integrating sphere in diffuse reflection was used to analyze the remaining samples. All samples were analyzed three times in a 50 mm diameter rotating cup. The percentage of crude protein (CP: nitrogen content × 6.25: Robbins, 1983) is an important limiting nutrient for large herbivores (Sinclair, 1975), and it was already used as an index of forage quality in *Rupicapra* species (cf. Corlatti & Bassano, 2014; Corlatti et al., 2013; Gálvez‐Cerón et al., 2013; Villamuelas et al., 2017). CP was thus assumed as an index of forage quality also in this study.

### 2.3 Ecological correlates

**FIGURE 1:**
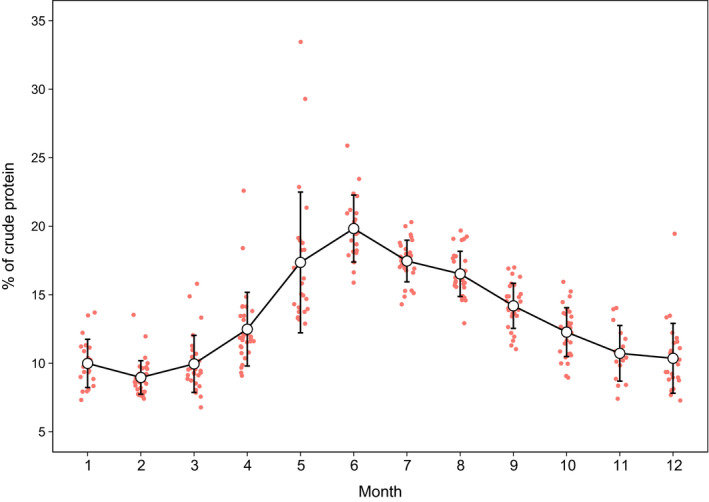
Monthly variation in percentage of fecal NIRS crude protein between 2011 and 2012 in male chamois within the Gran Paradiso National Park. The figure shows mean (open circles) ± *SD* (vertical bars). Datapoints are jittered to improve visualization

To investigate variation in percentage of CP (Figure 1), each fecal sample was linked with several ecological variables. Individual covariates such as age and mating behavior (i.e., territorial vs. nonterritorial: Corlatti et al., 2012, 2019) were excluded because this information would not be available when sampling is carried out anonymously or with DNA genotyping. As dietary quality can be affected by weather conditions (Halbritter & Bender, 2015), minimum air temperature (in °C), total precipitation (in mm), and snow depth (i.e., the depth of the new and old snow remaining on the ground at observation time, in cm) were considered as potential predictors of CP content; minimum and maximum air temperature were highly correlated and only the former was retained in the dataset. The effects of weather conditions can be investigated over different temporal scales. For example, Halbritter and Bender (2015) investigated the effect of mean precipitation one month prior to feces deposition, thus reflecting long‐term changes in forage quality. Climatic conditions, however, can also affect chamois daily feeding activity (Brivio et al., 2016; Mason, Stephens, Apollonio, & Willis, 2014), possibly influencing short‐time dietary selection (cf. Mason, Brivio, Stephens, Apollonio, & Grignolio, 2017). In this study, I investigated the relationship between fecal CP and environmental correlates on a daily basis, as this allows to match more naturally the timing of fecal sampling and weather data collection, while avoiding somewhat arbitrary definitions of long‐term weather effects. Assuming a retention time of *c*. 1 day for an ungulate the size of a chamois (cf. Illius & Gordon, 1992), weather data registered the day before feces deposition were retrieved from a meteorological station within the study site (Lago Serrù, 2,275 m a.s.l.). Additionally, the mean elevation of each individual the day before feces deposition was calculated from GPS‐collar data with at least three (2D) or four satellites (3D) and, respectively, DOP (dilution of precision) values lower than 5 and 10 (Lewis, Rachlow, Garton, & Vierling, 2007).

### 2.4 Statistical analysis

All analyses were conducted with R 3.6.1 (R Core Team, 2019) in RStudio 1.2.1335 (RStudio Team, 2019). To assess the importance of individual heterogeneity on CP variation, two Gaussian linear models were fitted within different environmentally and socially defined time periods, slightly modified from Corlatti (2018) to achieve more balanced sample size: year (January–December, *n* = 314), winter (January–March, *n* = 83), spring (April–May, *n* = 54), summer (June–September, *n* = 107), and autumn (October–December, *n* = 70). The first model was an “informed” linear mixed effect model fitted with the package “lmerTest” (Kuznetsova, Brockhoff, & Christensen, 2017), with period‐specific predictors plus animal identity as random factor, reflecting identifiable sampling (Equation 1). The second, a “naïve” linear model fitted with the “stats” package (R Core Team, 2019), with the same set of period‐specific predictors but without individual random effect, reflecting anonymous sampling and analysis of pseudoreplicated data (Equation 2).

(1) $$\begin{array}{c} {\text{CP}}_{\mathrm{ij}}∼N\left(\mu_{\mathrm{ij}},\sigma^{2}\right)\\ E\left({\text{CP}}_{\mathrm{ij}}\right)=\mu_{\mathrm{ij}}\;\text{and}\;\text{var}\left({\text{CP}}_{\mathrm{ij}}\right)=\sigma^{2}\\ \mu_{\mathrm{ij}}=X1_{\mathrm{ij}}+\cdots +Xn_{\mathrm{ij}}+{\text{Individual}}_{j}\\ {\text{Individual}}_{j}∼N\left(0,\sigma_{\text{Individual}}^{2}\right) \end{array}$$

(2) $$\begin{array}{c} {\text{CP}}_{i}∼N\left(\mu_{i},\sigma^{2}\right)\\ E\left({\text{CP}}_{i}\right)=\mu_{i}\;\text{and}\;\text{var}\left({\text{CP}}_{i}\right)=\sigma^{2}\\ \mu_{i}=X1_{i}+\cdots +Xn_{i} \end{array}$$

${\text{CP}}_{i(j)}$ was the value of crude protein for measure *i* (at individual *j*), log_10_‐transformed to approximate a symmetrical distribution. ${\text{Individual}}_{j}$ was the random factor, assumed to be normally distributed with mean 0 and variance $\sigma_{\text{Individual}}^{2}$ . $X1_{i(j)}+\cdots +Xn_{i(j)}$ were the standardized continuous predictors included in the models within each period. Minimum air temperature, total precipitation, and mean elevation were included in all models. Snow depth was included only in the winter, spring, and autumn models, because of absence of snow in summer and collinearity with minimum temperature over the year (*r_p_* > .7, Dormann et al., 2013). Models’ fit was assessed visually through residual diagnostics.

For all informed models I estimated: (a) The significance of the individual random intercept fitting exact likelihood ratio tests between the informed and the corresponding naïve models, with the package “RLRsim” (Scheipl, Greven, & Kuechenhoff, 2008); (b) the variance explained by the individual random effect, given by the difference between conditional and marginal *R* ^2^ statistics (Nakagawa & Schielzeth, 2013), with the package “MuMIn” (Bartoń, 2019); (c) the individual repeatability adjusted for predictors (Nakagawa & Schielzeth, 2010), with the “rptR” package (Stoffel, Nakagawa, & Schielzeth, 2017). Parameter estimates were checked for consistency between informed and naïve models within each period, to assess the consequences of anonymous sampling.

## 3 RESULTS

Residual diagnostics indicated no major violation of model assumptions. The coefficient of variation for individual sample size within different time periods was: 39% in the full dataset; 37% in winter; 43% in spring; 34% in summer; 44% in autumn. The likelihood ratio test was significant for the full dataset (LRT = 3.766, *p*‐value = .014), in winter (LRT = 23.955, *p*‐value < .001), spring (LRT = 2.364, *p*‐value = .049), and autumn (LRT = 2.481, *p*‐value = .047), but not in summer (LRT = 0.324, *p*‐value = .191). The variance explained by the individual random effect was: 3.2% in the full dataset; 47.1% in winter; 13.2% in spring; 4.6% in summer; 18.7% in autumn. Adjusted repeatability values ± *SE* were: 0.06 ± 0.04 in the full dataset, 0.57 ± 0.11 in winter, 0.24 ± 0.16 in spring, 0.05 ± 0.07 in summer, and 0.26 ± 0.14 in autumn. The parameter estimates of the informed and naïve models were broadly very similar in all time periods (Table 1). Dietary quality had a significant positive relationship with temperature over the year, in winter and in autumn. Snow affected negatively the quality of diet in winter and in spring, and a negative relationship was detected between diet quality and elevation in spring and in summer (Table 1).

**TABLE 1:** Parameter estimates of informed (mixed effect) and naïve linear models fitted to investigate the consequences of identifiable versus anonymous sampling in f‐NIRS analysis in chamois, within the Gran Paradiso National Park between 2011 and 2012

	Informed models			Naïve models		
	Estimate	St. Err.	*p*‐Value	Estimate	St. Err.	*p*‐Value
Year						
Intercept	1.105	0.007	<.001	1.103	0.005	<.001
Temp. min	**0.091**	**0.007**	**<.001**	**0.091**	**0.006**	**<.001**
Precipitation	−0.000	0.005	.975	−0.000	0.005	.956
Elevation	−0.000	0.007	.951	−0.003	0.006	.657
Winter						
Intercept	0.977	0.013	<.001	0.973	0.008	<.001
Temp. min	**0.020**	**0.006**	**.001**	**0.018**	**0.008**	**.025**
Precipitation	−0.008	0.007	.244	−0.005	0.009	.587
Snow	**−0.019**	**0.006**	**.004**	**−0.018**	**0.009**	**.046**
Elevation	−0.015	0.009	.082	−0.001	0.008	.920
Spring						
Intercept	1.152	0.015	<.001	1.149	0.12	<.001
Temp. min	0.008	0.012	.553	0.002	0.013	.886
Precipitation	−0.020	0.012	.110	−0.023	0.013	.070
Snow	**−0.080**	**0.012**	**<.001**	**−0.077**	**0.013**	**<.001**
Elevation	**−0.032**	**0.014**	**.028**	**−0.031**	**0.013**	**.026**
Summer						
Intercept	1.220	0.007	<.001	1.219	0.006	<.001
Temp. min	−0.002	0.007	.739	−0.002	0.007	.794
Precipitation	−0.004	0.007	.514	−0.005	0.007	.501
Elevation	**−0.015**	**0.007**	**.042**	**−0.015**	**0.007**	**.020**
Autumn						
Intercept	1.045	0.012	<.001	1.043	0.009	<.001
Temp. min	**0.037**	**0.012**	**.004**	**0.035**	**0.013**	**.011**
Precipitation	−0.003	0.009	.727	−0.004	0.009	.681
Snow	−0.012	0.013	.331	−0.012	0.013	.359
Elevation	0.003	0.013	.851	0.002	0.012	.842

Note

The table reports parameter estimates, standard errors, and *p*‐values calculated using Satterthwaite approximation. Significant predictor estimates are shown in bold.

## 4 DISCUSSION

Despite the use of anonymous fecal sampling is widespread in wildlife nutritional ecology (e.g., Gad & Shyama, 2011; Gálvez‐Cerón et al., 2013; Halbritter & Bender, 2015), to date no information was available about the effects of neglecting individual variation in studies of dietary quality. Individual repeatability in chamois dietary quality was highest in winter and lowest in summer, and the variance explained by the individual random effect generally reduced when looking at the full data set as compared to the seasonal estimates. In all time periods, estimates of dietary quality correlates were unaffected by the removal of individual variation. This suggests that pseudoreplication deriving from anonymous fecal sampling was inconsequential.

Individual trait variation is ubiquitous in wildlife populations, and the study of individual heterogeneity offers invaluable opportunities to improve our understanding of the trade‐off patterns in life history traits (Harper, 1994; Hayes & Jenkins, 1997). Different allocation of energy and nutrients to the tissues and the activities and time required for survival, growth, and reproduction may in fact generate from individual differences at multiple levels, including sex, age, personalities, space use, and environmental conditions experienced over a lifetime (cf. Douhard et al., 2014; Emlen, 1970; Gimenez, Cam, & Gaillard, 2018; Nakayama, Rapp, & Arlinghaus, 2017). As a result, the importance of variation within and between individuals in shaping ecological processes is increasingly appreciated in many fields of research such as demography (Gimenez et al., 2018), stress physiology (Taff, Schoenle, & Vitousek, 2018), and nutritional ecology (Steyaert et al., 2012). Furthermore, failing to include individual heterogeneity when modeling variation in the trait under study can mislead interpretations of ecological patterns (Coppes et al., 2018; Hamel, Côté, Gaillard, & Festa‐Bianchet, 2009; Richard, Toïgo, Appolinaire, Loison, & Garel, 2017). The choice of modeling individual variation is thus always desirable, as it allows to simultaneously gain insights into ecological processes and address issues of pseudoreplication.

The costs for individual identification, however, may be important and understanding the consequences of neglecting individual heterogeneity provides useful information to optimize sampling designs (cf. Coppes et al., 2018; Corlatti, 2018). This study supports the use of anonymous fecal sampling in studies of chamois nutritional ecology. Extending this result to other taxa, however, requires caution. Similar results were obtained when the ecological correlates of fecal cortisol metabolites (FCMs) were investigated in chamois (Corlatti, 2018), but FCM studies on species with faster life histories (i.e., snowshoe hare *Lepus timidus*, capercaillie *Tetrao urugallus*), highlighted the importance of accounting for individual heterogeneity to obtain robust estimates (Coppes et al., 2018; Rehnus & Palme, 2017). Clarifying if individual consistency in dietary quality reflects the slow‐fast continuum in life histories (i.e., lowest in long‐lived species, highest in short‐lived ones, cf. Gaillard et al., 2016), as observed in other traits (Nakayama et al., 2017; Péron et al., 2016), might help to understand if this result can be extended to taxa with life histories similar to the chamois'.

It is worth noting, however, that no hard rules exist on how large the intraclass correlation coefficient should be to proclaim consequential or inconsequential lack of independence. This is especially true when the intraclass correlation coefficient is estimated as adjusted repeatability (Nakagawa & Schielzeth, 2010). Predictors associated with individual data points (e.g., age over different years) will usually increase repeatability estimates because they will reduce residual variance within individuals, whereas predictors that vary between individuals (e.g., sex) will usually decrease repeatability because they will reduce variance among individuals (Gelman & Hill, 2007). The nature of adjusted repeatability is thus intrinsically relative. The period of data collection may also have an impact on the importance of individual variation, likely because in different periods animals must face different constraints, thus have different opportunities for expressing repeatable among‐individual differences.

In mountain areas, temperature is strongly collinear with Julian date, and the observed positive relationship between minimum temperature and dietary quality over the year likely reflected seasonality in primary production (Pettorelli, Pelletier, von Hardenberg, Festa‐Bianchet, & Côté, 2007). Seasonality makes it difficult to maintain consistent dietary quality within individuals and consistent dietary differences among individuals. With increasing availability of food resources, a given individual has greater possibilities to access food items of different quality; at the same time, competition for food is relaxed and different individuals might have greater possibilities to select food items of similar quality. This, in turn would explain the reduction in repeatability, among‐individual heterogeneity and variance in the full year and in summer as compared to the other seasons. Conversely, in winter, repeatability, among‐individual heterogeneity and variance were greatest. Given the low availability of food resources in this time of the year, it seems plausible that the possibility of selecting food of different quality declines, while among‐individual competition for food increases. Since individuals have different abilities to access high quality food resources when forage availability declines (cf. Fattorini et al., 2018), repeatability and among‐individual heterogeneity and variance in winter dietary quality may be expected to increase. Furthermore, in winter and spring, decreasing temperature and increasing snow depth tend to hamper chamois daily activity (Brivio et al., 2016). My data suggest that this conservative strategy may be traded‐off against lower quality of food: with high snow cover and low temperatures, chamois may spend little time feeding and thus settle for lower‐quality food, as compared to days when milder temperatures and lower snow cover allow for higher selectivity. The negative relationship between elevation and dietary quality in spring and summer is somewhat surprising, as CP typically increases with altitude (Albon & Langvatn, 1992). However, this effect may be confounded by unmodelled individual variation in elevation used at different time of day. In Alpine ibex *Capra ibex*, for example, animals in summer tend to stay at higher elevation during daylight hours but feed at lower elevations in the evening (Aublet, Festa‐Bianchet, Bergero, & Bassano, 2009), making the effect of altitude on dietary quality weak. In addition, the negative relationship observed in this study might also be confounded by different foraging abilities of territorial and nonterritorial males, which in summer occupy significantly different elevations (Corlatti et al., 2013).

When pseudoreplication occurs, several remedies can be applied either at the sampling stage or during data analysis (Millar & Anderson, 2004), but they typically assume domain over the source of nonindependence (cf. Hurlbert, 1984). The problem of anonymous sampling is that the source of nonindependence is known (the individual), but impossible to control for. To mitigate the issue of pseudoreplication, feces collection should be sufficiently dispersed in space and in time to avoid resampling of individuals (Coppes et al., 2018). This “cautionary” sampling approach may effectively reduce pseudoreplication, although it requires some knowledge of the spatio‐temporal behavior of the target species, and its efficacy depends on other factors such as population density (in small populations the risk of pseudoreplicates increases). Recently, analytical remedies for pseudoreplication when sampling is unknown have been proposed. For example, individual identities could be randomly assigned with replacement to each fecal sample, so that “randomly informed” multilevel models can be used to estimate covariate parameters (Garamszegi, 2016). Alternatively, the spatial or temporal autocorrelation in the response variable could be considered (Garamszegi, 2016). The latter solution requires reliable knowledge of the spatio‐temporal behavior of the target species, whereas the former appears more widely applicable. Simulation studies, however, showed that random assignment is ineffective at resolving nonindependence and basically reduces to a naïve model (Garamszegi, 2019; Gratton & Mundry, 2019). My dataset is not ideal to test the random assignment method, as the estimates of informed and naïve models are similar. However, preliminary analyses conducted on the winter dataset support the conclusion of Gratton and Mundry (2019) and Garamszegi (2019). Anonymous fecal sampling in studies of dietary quality may represent an opportunity to optimize the trade‐offs between costs and benefits of different sampling strategies when dietary quality is not highly consistent. When pseudoreplication is consequential, however, no conclusive remedy exists to resolve nonindependence, and identifiable sampling is required to obtain robust estimates.

## CONFLICT OF INTERESTS

I have no competing interests.

## AUTHOR CONTRIBUTION

**Luca Corlatti:** Conceptualization (lead); Data curation (lead); Formal analysis (lead); Investigation (lead); Methodology (lead); Writing‐original draft (lead); Writing‐review & editing (lead).

## Data Availability

Data used in this analysis are available at Dryad Digital Repository: https://doi.org/10.5061/dryad.tht76hdwn.
